# The where, what, and when of membrane protein degradation in neurons

**DOI:** 10.1002/dneu.22534

**Published:** 2017-09-19

**Authors:** Eugene Jennifer Jin, Ferdi Ridvan Kiral, Peter Robin Hiesinger

**Affiliations:** ^1^ Division of Neurobiology Institute for Biology, Freie Universität Berlin 14195 Berlin Germany; ^2^ Graduate School of Biomedical Sciences University of Texas Southwestern Medical Center Dallas TX 75390 USA

**Keywords:** neuronal maintenance, membrane degradation, endosome, lysosome, autophagy

## Abstract

Membrane protein turnover and degradation are required for the function and health of all cells. Neurons may live for the entire lifetime of an organism and are highly polarized cells with spatially segregated axonal and dendritic compartments. Both longevity and morphological complexity represent challenges for regulated membrane protein degradation. To investigate how neurons cope with these challenges, an increasing number of recent studies investigated local, cargo‐specific protein sorting, and degradation at axon terminals and in dendritic processes. In this review, we explore the current answers to the ensuing questions of where, what, and when membrane proteins are degraded in neurons. © 2017 The Authors Developmental Neurobiology Published by Wiley Periodicals, Inc. Develop Neurobiol 78: 283–297, 2018

## INTRODUCTION

Continuous synthesis and degradation through homeostatic regulation of protein turnover ensure a functional pool of proteins. Neuronal longevity and morphological complexity represent challenges for both cytosolic and membrane protein turnover (Steward and Schuman, 2003; Wang and Hiesinger, 2012; Bezprozvanny and Hiesinger, 2013; Alvarez‐Castelao and Schuman, 2015). Cytosolic proteins are predominantly subject to proteasomal degradation (Ciechanover, 2005; Yi and Ehlers, 2007; Tai and Schuman, 2008; Bhattacharyya et al., 2014; Cohen‐Kaplan et al., 2016). In contrast, membrane proteins are either degraded through an endo‐lysosomal mechanism or autophagy (Klionsky and Emr, 2000; Luzio et al., 2007; Eskelinen and Saftig, 2009; Schulze et al., 2009; Tooze et al., 2014; Huber and Teis, 2016; Galluzzi et al., 2017). Defects in cytosolic and membrane protein degradation typically result in protein accumulation and neuronal dysfunction. Such defects can occur at synapses prior to defects in the cell body and are hallmarks of many neurodegenerative diseases (Arendt, 2009; Shankar and Walsh, 2009; Wong and Cuervo, 2010; Morales et al., 2015; Soto and Kerschensteiner, 2015).

Local protein synthesis and degradation via the proteasome have long been described in neurons (Steward and Schuman, 2003). Recent whole‐proteome analyses in yeast suggest distinct subcellular localization of protein synthesis and degradation pathways, which might indicate an evolutionary base for the compartmentalized regulation of these events in morphologically more complex cells, including neurons (Shao and Hegde, 2014). Interestingly, a recent proteomics study in neurons based on inhibition of the ubiquitin‐proteasome system indicated that only a minority of synaptic proteins depend on proteasomal degradation under basal conditions (Hakim et al., 2016). However, many of the proteins for which local mRNA deposits have been found in dendrites are membrane proteins, including the NMDA and inositol 1,4,5‐triphosphate (InsP3) receptors (Steward and Schuman, 2003). Here, we focus on membrane degradation and refer readers to excellent reviews on the degradation of cytoplasmic proteins by the ubiquitin‐proteasome system (Bingol and Schuman, 2005; Yi and Ehlers, 2007; Tai and Schuman, 2008; Alvarez‐Castelao and Schuman, 2015; Kaushik and Cuervo, 2015; Labbadia and Morimoto, 2015; Cohen and Ziv, 2017).

Membrane protein turnover is of particular importance to the maintenance of neuronal function. At the presynaptic terminal the synaptic vesicle cycle poses a formidable challenge to membrane protein turnover. Recent work has provided evidence for how dysfunctional or aging synaptic proteins are sorted for degradation (Uytterhoeven et al., 2011; Fernandes et al., 2014; Sheehan et al., 2016). Similarly, the postsynaptic compartment requires continuous cycles of endocytosis/exocytosis of membrane proteins, such as neurotransmitter receptors (Coussen, 2009; Santos et al., 2009). However, where the actual degradation occurs, with what cargo‐specificity, and when during the development, function, and aging of neurons remain challenging questions for all known mechanisms.

Canonical endolysosomal degradation and autophagy are responsible for degradation of (but not limited to) membrane proteins. In both pathways, proteins are delivered to highly acidic, degradative organelles where degradation is initiated by acidification‐activated proteases (Kaur and Debnath, 2015; Xu and Ren, 2015; Luzio et al., 2007; Schink et al., 2016; Takats et al., 2016; Lorincz et al., 2017). Canonical endolysosomal degradation requires maturation of early endosome to late endosome or multivesicular body (MVB), followed by fusion with lysosomes for degradation. Ubiquitin attachment to membrane proteins serves as a signal for the endocytic internalization from the plasma membrane and is the signal for trafficking of protein from early endocytic vesicles to MVBs. Endosomal sorting complexes required for transport (ESCRT) is the core protein machinery to recognize ubiquitinated proteins in endosomes and sort them to MVBs (Katzmann et al., 2001; Henne et al., 2011).

In (macro‐) autophagy, a double membrane called phagophore forms around “to‐be‐degraded” cargo, such as vesicles containing membrane proteins, cytosolic proteins, protein aggregates, and organelles. After cargo engulfment, autophagosomes fuse with lysosomal vesicles to form degradative autolysosomes (Xie and Klionsky, 2007; Kraft and Martens, 2012; Coutts and La Thangue, 2016; Nishimura et al., 2017; Wang et al., 2017). Despite the abundance of transmembrane proteins in both presynaptic and postsynaptic compartments, progress has only recently been made to address what membrane proteins are degraded by either mechanism in neurons (Ashrafi and Schwarz, 2013; Huber and Teis, 2016; Mancias and Kimmelman, 2016; Zaffagnini and Martens, 2016; Vijayan and Verstreken, 2017). More studies have focused on the dysregulation of these degradative pathways in relation to neurodegenerative diseases than their wildtype maintenance function (Hara et al., 2006; Komatsu et al., 2006; Menzies et al., 2017; Moors et al., 2017). In the following sections, we will focus on membrane degradation via the autophagosomal and endolysosomal system, and our current understanding of where, what, and when these mechanisms degrade membrane proteins in neurons independent of disease‐specific neurotoxic insults.

## WHERE ARE MEMBRANE PROTEINS DEGRADED IN NEURONS?

**Figure 1:**
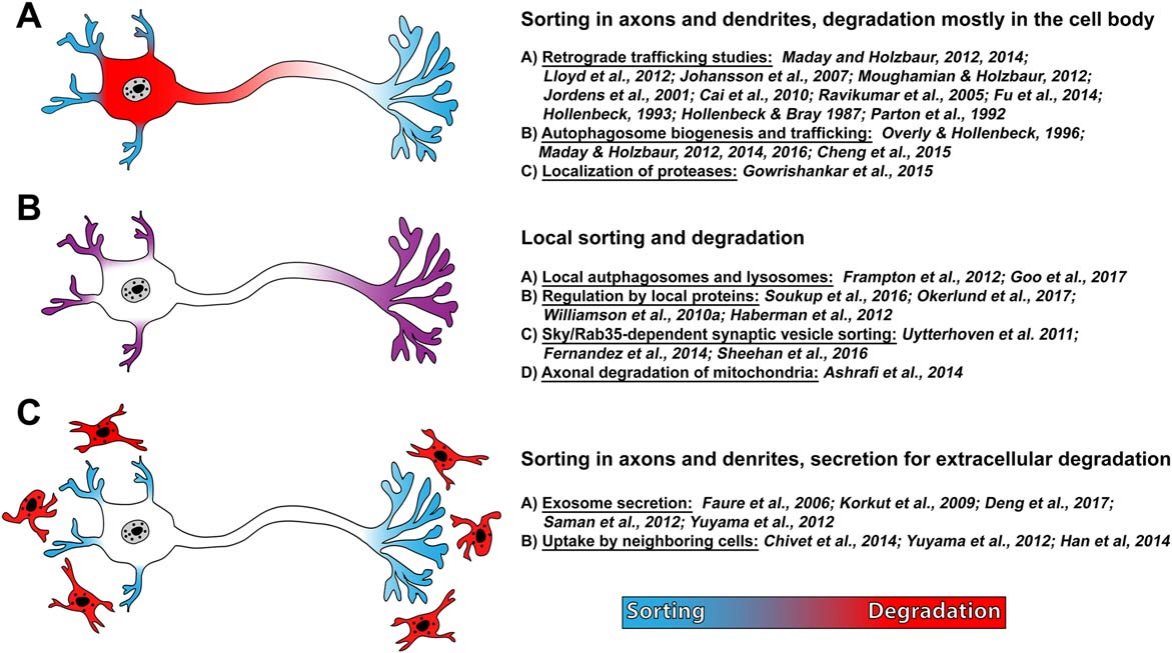
Three models for where neuronal membrane proteins sort and degrade. (A) Synaptic membrane proteins are sorted for degradation locally, and then undergo retrograde axonal trafficking to the cell body for degradation. (B) Sorting and degradation of synaptic membrane proteins occur locally in axon terminals and dendrites. (C) Neurons release synaptic membrane proteins outside via extracellular vesicles, which are taken by neighboring cells for degradation.

The distances between dendrites, the cell body, and the axon tip raise questions about the spatial regulation of membrane protein sorting and degradation. Three ways to remove synaptic membrane proteins from distal axons and dendrites have been proposed (Fig. 1): (1) synaptic membrane proteins may be sorted into endosomes and autophagosomes for retrograde trafficking back to the cell body, (2) membrane proteins may be sorted and degraded locally, and (3) membrane proteins may be secreted and taken up by neighboring cells for degradation. In this section, we will review current evidence for these three possibilities. How the implicated endolysosomal or autophagosomal mechanisms may operate in the different neuronal compartments will be discussed in the subsequent section.

### Retrograde Transport Back to Cell Body

Neurons employ robust microtubule‐dependent transport machinery to traffic proteins and organelles between the cell body, dendrites and distal axons (Maday et al., 2014). Microtubule‐based axonal transport utilizes two main types of molecular motors: kinesin for plus‐end directed anterograde transport (from cell body to axon terminals) and dynein‐dynactin complex for minus‐end directed retrograde transport (from axon terminals to cell body) (Schnapp and Reese, 1989; Pilling et al., 2006; Encalada et al., 2011). Live imaging of late endosomes, lysosomes, and autophagosomes in axons revealed net retrograde trafficking, suggesting that degradation may happen in the cell body [Fig. 1(A)] (Hollenbeck and Bray, 1987; Parton et al., 1992; Hollenbeck, 1993; Maday et al., 2014; Cheng et al., 2015). Defective retrograde transport causes dramatic accumulations of late endosomes and autophagosomes in axons as well as impaired autophagic and lysosomal degradation (Ravikumar et al., 2005; Cai et al., 2010; Lloyd et al., 2012). Such defects have been linked to late‐onset, progressive motor neuron degeneration such as amyotrophic lateral sclerosis (ALS) or spinal muscular atrophy (SMA) (Hafezparast et al., 2003; Puls et al., 2003; Munch et al., 2004; Levy et al., 2006; Lai et al., 2007; Chevalier‐Larsen et al., 2008; Laird et al., 2008; Strom et al., 2008; Hirokawa et al., 2010).

Autophagosomes form in the distal axons, initially exhibit bidirectional movements, but eventually switch to robust retrograde transport for degradation in the cell body (Maday and Holzbaur, 2012, 2014; Maday et al., 2014). This initial bidirectional transport is carried out when both kinesin‐1 and dynein motors tightly bind to autophagosomes, but on interaction between LC3 on autophagosome and JNK‐interacting protein 1 (JIP1), kinesin‐1 activation is blocked, resulting in the robust retrograde transport back to cell body (Fu et al., 2014; Maday et al., 2014). Dynein motors are recruited to autophagosomes after fusion with late endosomes (Cheng et al., 2015). In some studies, autophagosome maturation and degradation were observed only during or after retrograde transport following autophagosome biogenesis at the axon terminal (Maday and Holzbaur, 2012), consistent with the earlier report of the progressive increase in the proportion of acidic endocytic organelles along axons closer to the soma (Overly and Hollenbeck, 1996). These findings support the prevalent idea that degradation may occur preferentially in the cell body or en route to the cell body [Fig. 1(A)] (Maday and Holzbaur, 2016).

Late endosomes and lysosomes exhibit retrograde transport behavior distinct from autophagosomes, most likely resulting from different molecular interactions with adaptors and motor proteins. Kinesin‐2 is the primary motor protein to regulate anterograde movement of late endosomes and lysosomes (Brown et al., 2005). Retrograde transport of late endosomes and lysosomes requires direct interaction between Rab7 effector protein Rab7 Interacting Lysosomal Protein (RILP) and the C‐terminal region of a dynactin subunit p150 (Glued) (Jordens et al., 2001; Johansson et al., 2007; Maday et al., 2014). The highly conserved CAP‐Gly domain of p150 initiates the retrograde transport from distal axons (Lloyd et al., 2012; Moughamian and Holzbaur, 2012). Snapin, a neuronal SNARE‐binding protein, also regulates retrograde transport of late endosomes by tethering them to dynein (Cai et al., 2010). Unlike autophagosomes that exhibit robust retrograde transport, late endosomes, and lysosomes have been reported to traffic bidirectionally with frequent pauses and directional changes in a constant tug‐of‐war between the opposing motors that are simultaneously present on the organelles (Deacon et al., 2003; Bananis et al., 2004; Muller et al., 2008; Hendricks et al., 2010; Lloyd et al., 2012; Maday et al., 2014).

Lysosomes containing proteases are preferentially enriched in the cell body, whereas lysosomes at distal axons have been reported to lack luminal proteases and, therefore, lack degradative capacity (Gowrishankar et al., 2015). In this case, late endosomes, lysosomes, and autophagosomes would require to retrogradely traffic back to cell body for degradation [Fig. 1(A)]. Highly acidic pH is required for lysosomal function, and less acidic pH can, therefore, reduce the degradative capacity of lysosomes. A recent study reported that lysosomal pH depends on intracellular positioning, with lysosomes closer to the cell periphery being less acidic (Johnson et al., 2016), although it is not known if this is also the case for lysosomes at synapses. These studies support the need for retrograde transport of degradative organelles and degradation in the cell body.

In some vertebrates, synapses can be separated from the cell body through axons over a meter in length. Considering these distances, even if the cargos are transported at the fastest reported axonal speed (∼10 cm/day), it would take days for synaptic proteins to reach the cell body for degradation (Grafstein and Forman, 1980; Miller and Heidemann, 2008). Such long distances may not be an obstacle for retrograde trafficking‐dependent degradation if dysfunctional cargoes do not affect normal function and health of a neuron. Alternatively, degradation and protein synthesis are often closely linked through feedback mechanisms that ensure protein homeostasis (Alvarez‐Castelao and Schuman, 2015; Cajigas et al., 2010). In this case, long trafficking distances would represent a challenge for mechanisms of local protein homeostasis in neurons.

### Local Membrane Protein Degradation at Synapses

Neurons have to rapidly and homeostatically respond to varying conditions both during developmental axonal and dendritic growth as well as neuronal activity. Ample evidence for local protein synthesis and proteasomal degradation in dendrites strongly suggest the autonomous regulation of protein turnover at synapses (Pierce et al., 2001; Wang et al., 2010; Ramirez and Couve, 2011; Holt and Schuman, 2013). Most studies on protein degradation in neurons have focused on proteasomal degradation, and several studies have demonstrated that proteins are degraded locally through proteasomal degradation at postsynaptic terminals (Speese et al., 2003; Yi and Ehlers, 2007; Hamilton and Zito, 2013). However, proteasomal degradation may not be responsible for the degradation of the majority of synaptic proteins in axon terminals (Hakim et al., 2016).

Similar to the proteasomal degradation machinery, membrane degradation machinery has been observed both at axon terminals and near or in dendritic spines (Frampton et al., 2012; Goo et al., 2017). Frampton et al. (2012) showed the abundance of proteins lysosomal‐associated membrane protein 2 (LAMP2) and microtubule‐associated protein 1 light chain 3 (LC3) in axons and axon terminals, suggesting that lysosomes and autolysosomes are located at axon terminals. Using compartmentalized rat superior cervical ganglion (SCG) neuronal cultures, they observed a significant increase of LAMP2 protein exclusively in the distal axons on nerve growth factor (NGF) stimulation (Frampton et al., 2012). Recruitment of lysosomes to dendritic spines in an activity‐dependent manner was also recently reported (Goo et al., 2017). These observations suggest that the abundance of lysosomes, and perhaps lysosomal degradation, is regulated locally at distal axon terminals, and possibly independently from lysosomes at the cell body [Fig. 1(B)].

**Figure 2:**
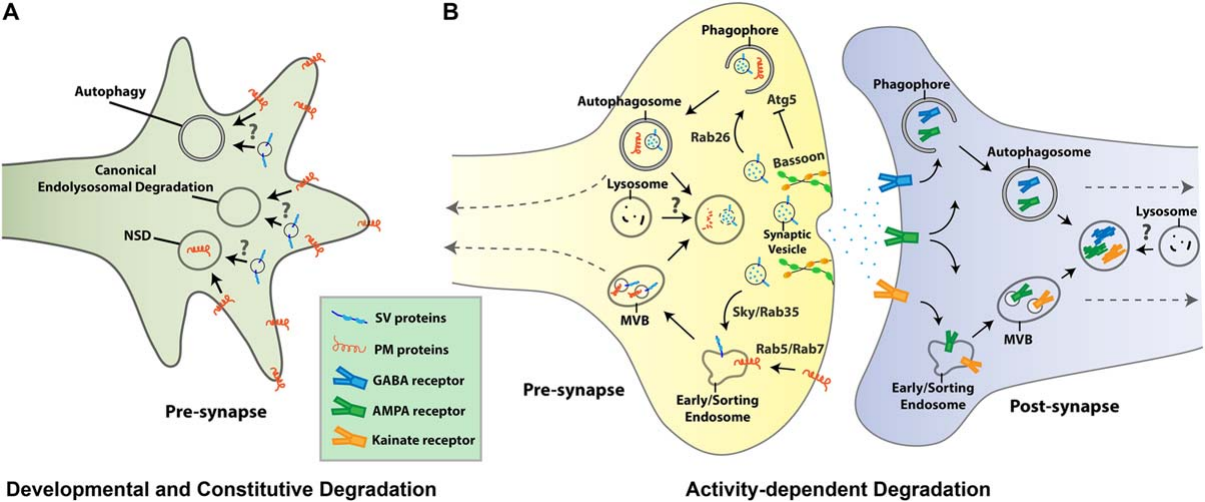
Schematic overviews of cargo‐sorting and degradation mechanisms operating in developing and mature neurons. (A) Developmental and constitutive degradation by autophagy, canonical endolysosomal degradation, and neuron‐specific endolysosomal degradation (Neuronal Sort and Degrade; NSD). Plasma membrane (PM) proteins such as guidance receptors are degraded through NSD (Williamson et al., 2010b). It has not been shown what membrane proteins are degraded through autophagy and canonical endolysosomal degradation in a developing neuron. (B) Mechanisms of activity‐dependent cargo‐sorting and degradation in presynaptic and postsynaptic terminals through endolysosomal degradation or autophagy.

Several recent studies characterized mechanisms for the local regulation of autophagosome formation by synaptic proteins at axon terminals. Soukup et al. (2016) demonstrated an unexpected role of the synaptically enriched protein Endophilin A (EndoA), which was previously characterized during synaptic vesicle endocytosis (Song and Zinsmaier, 2003). On phosphorylation by the Parkinson's disease‐associated kinase LRRK2, EndoA recruits Atg3 to the growing membrane of autophagosomes and causes it to colocalize with Atg8 (LC3) (Soukup et al., 2016). In addition, Okerlund et al. (2017) showed that the presynaptic active zone protein Bassoon selectively inhibits autophagy by interacting with Atg5 [Fig. 2(B)]. The CC2 domain of Bassoon interacts with Atg5, possibly regulating the formation of the Atg5‐Atg12‐Atg16 complex. Loss of Bassoon function triggers synaptic autophagy (Okerlund et al., 2017). The idea of autophagosome formation at the axon terminals is consistent with findings by Maday and Holzbaur (2012, 2014, 2016). Although local degradation of membrane proteins via autophagy has not been directly demonstrated, local degradation of mitochondria through PINK/PARKIN‐mediated mitophagy at axon terminals has been reported (Ashrafi et al., 2014). As shown in this study, selective induction of mitochondrial damage by mitochondrial KillerRed (mt‐Kr), a genetically encoded photosensitizer targeted to mitochondria, leads to the recruitment of autophagosomes and lysosomes to damaged mitochondria for degradation locally in the axons.

In addition to autophagy, two neuron‐specific proteins that predominantly function at synaptic terminals have been reported to constitute a local, neuron‐specific branch of the endolysosomal degradation system: the vesicular ATPase component V100 and the vesicle SNARE neuronal Synaptobrevin (n‐Syb) (Williamson et al., 2010a; Haberman et al., 2012; Wang and Hiesinger, 2012; Bezprozvanny and Hiesinger, 2013). Both proteins were initially characterized as synaptic vesicle proteins in synaptic vesicle exocytosis (Perin et al., 1991; DiAntonio et al., 1993; Deitcher et al., 1998; Schoch et al., 2001; Hiesinger et al., 2005). Loss of function of either V100 or n‐Syb causes local sorting and degradation defects at axon terminals, which eventually lead to adult‐onset neurodegeneration in *Drosophila* photoreceptors (Williamson et al., 2010a; Haberman et al., 2012). After the initial cargo overload in endosomes in both mutants, autophagy is activated as an apparent secondary, compensatory effect. It is interesting to note that both proteins are almost exclusively located at axon terminals, indicating that neurons implement an additional, neuron‐specific endolysosomal sorting, and degradation mechanism locally at axon terminals to meet a high or specialized demand for membrane protein turnover.

Presence of membrane protein degradation machinery and identification and characterization of synaptic autophagy and endolysosomal degradation are strong indicators that membrane proteins are sorted for degradation locally at synapses [Fig. 1(B)]. However, direct demonstration of where membrane proteins are degraded in neurons remains unclear because protein degradation is mostly assayed in the entire neuron without separation of distal axons and dendrites from cell bodies (Cohen et al., 2013; Price et al., 2010; Sheehan et al., 2016; Cohen and Ziv, 2017). Protein half‐lives in neurons are studied using isotopic labeling with amino acids, such as stable isotope labelling with amino acids in cell culture (SILAC), followed by mass spectrometry (MS) in mouse or rat cortical neurons (Price et al., 2010; Cohen et al., 2013). Activity‐dependent protein degradation in neurons has been demonstrated biochemically with western blot analyses before and after neural activation in cultured rat hippocampal or cortical neuron (Shehata et al., 2012; Widagdo et al., 2015; Sheehan et al., 2016). Separation of neuronal compartments can be achieved using microfluidic culture devices (Taylor et al., 2005; Park et al., 2006; Taylor et al., 2006; Taylor and Jeon, 2010; Taylor et al., 2010). Such methods have been used to demonstrate local proteasomal degradation in growth cones (Deglincerti et al., 2015), and local degradation of damaged mitochondria by autophagy in axons (Ashrafi et al., 2014).

### Release into Neighboring Cells

Neurons can rid themselves of membrane proteins and other cargo through the release of extracellular vesicles (EVs) followed by uptake in a neighboring cell [Fig. 1(C)] (Simons and Raposo, 2009; Raposo and Stoorvogel, 2013; Budnik et al., 2016). Two major types of EVs are exosomes and microvesicles. Exosomes are intraluminal vesicles (ILVs) inside multivesicular bodies (MVBs) that are released into the extracellular space on direct fusion of MVBs with the plasma membrane. Microvesicles are vesicles that form by an outward budding of the plasma membrane. As a result, microvesicles are typically larger (100 nm to 1 um in diameter) than exosomes (30–100 nm in diameter) (Colombo et al., 2014). The content that can be released as EVs is not limited to membrane proteins and also includes mRNA, cytosolic proteins, and lipids. Two general functions of exosome secretion have been proposed: degradation and intercellular communication. Here, we focus on what is known about degradation of membrane proteins by neighboring cells, while other functions of exosomes are presented and comprehensively reviewed elsewhere (Lotvall and Valadi, 2007; Simons and Raposo, 2009; Fruhbeis et al., 2012; Raposo and Stoorvogel, 2013; Budnik et al., 2016).

Exosome secretion from neurons was first reported in mature cortical neurons as well as *in vivo* at the Drosophila neuromuscular junction (NMJ) (Faure et al., 2006; Korkut et al., 2009). Since then, many studies have focused on the release of disease‐associated proteins and aggregates, such as tau, amyloid‐beta peptides, huntingtin, and superoxide dismutase‐1 (SOD‐1), from neurons via exosomes (Saman et al., 2012; Yuyama et al., 2012; Deng et al., 2017). It was recently demonstrated that the synaptic vesicle protein cysteine string protein (CSPα) mediates the release of exosomes containing polyglutamine expanded protein 72Q huntingtin and superoxide dismutase‐1 (SOD‐1) (Deng et al., 2017). Consequently, loss‐of‐function of CSPα in *Drosophila* and *C. elegans* mutants demonstrate uncoordinated movements, neurodegeneration, and early lethality possibly due to defective release of 72Q huntingtin and SOD‐1 (Zinsmaier et al., 1994; Kashyap et al., 2014).

EVs released from a neuron can be taken up by another neuron, glia or any other neighboring cells. Synaptic activity was shown to bias the binding of exosomes to neighboring neurons rather than glial cells (Chivet et al., 2014). The EVs can then be degraded in any of these recipient cells by fusing directly with the plasma membrane followed by endocytosis and endolysosomal degradation. Amyloid‐beta peptides released via neuronal exosomes were reported to be cleared via microglia (Yuyama et al., 2012). Exosomes may also be released into other neighboring cells, including muscles at the neuromuscular junction (NMJ) (Budnik et al., 2016) and epithelial cells in the case of *Drosophila* sensory class IV dendritic arborization (da) neurons (Han et al., 2014). Furthermore, clearance of degenerating dendrite fragments of *Drosophila* class IV da neuron was reported for the neighboring epidermal cells (Han et al., 2014). In contrast, it is less clear whether synaptic membrane proteins are degraded in neighboring cells.

## WHAT MEMBRANE PROTEINS AND MECHANISMS ARE IMPLICATED IN NEURONAL DEGRADATION

Autophagosomes and endolysosomal compartments are available in distal axons and dendrites. Recent studies have started to identify what cargos may be degraded by these mechanisms, but the list of membrane proteins tested so far is relatively short (Fig. 2). In this section, we will discuss what is currently known about what cargoes are degraded by what membrane protein degradation mechanism in neurons.

### Synaptic Autophagy

Autophagy is highly conserved from yeast to mammals, and most of our knowledge is derived from studies in non‐neuronal cells or neuronal cell bodies (Ohsumi, 2014). Unlike non‐neuronal cells, primary cultured neurons may not upregulate autophagy in response to starvation, but instead employ constitutive autophagy to maintain protein homeostasis (Maday and Holzbaur, 2016). Several recent studies focused on the molecular characterization of neuronal autophagy, particularly at presynaptic terminals (Soukup et al., 2016; Okerlund et al., 2017; Vanhauwaert et al., 2017; Vijayan and Verstreken, 2017).

Macroautophagy is well characterized for its ability to bulk degrade membrane proteins, organelles, cytosolic proteins, and protein aggregates. The recent observation of synaptic vesicles in pre‐autophagosomal structures in *Drosophila* NMJ and cultured rat hippocampal neuron cell bodies by Binotti et al. (2015) suggests that synaptic vesicle proteins may be degraded as bulk cargo, although degradation was not directly shown [Fig. 2(B)]. They demonstrated that a small Rab GTPase Rab26, which is predominantly localized to synaptic regions in *Drosophila* (Chan et al., 2011), interacts with Atg16L and directs synaptic vesicles to pre‐autophagosomal structures (Binotti et al., 2015). Rab26 overexpression resulted in accumulation of synaptic vesicles in preautophagosomes in *Drosophila* NMJ. This suggests a mechanism that directly engulfs the entire synaptic vesicles for degradation without more specific protein sorting.

Postsynaptic membrane proteins such as GABA and AMPA receptor subunits are reported to be degraded through autophagy, although where these receptors are degraded still remains unknown [Fig. 2(B)] (Rowland et al., 2006; Shehata et al., 2012; Widagdo et al., 2015). Degradation of AMPA receptor subunits GluA1 and GluA2, assayed by western blot analyses of cultured rat hippocampal or cortical neurons, was correlated with the autophagosomal or lysosomal markers LC3‐II and LAMP1, respectively (Shehata et al., 2012; Widagdo et al., 2015). Moreover, inhibition of autophagy with Wortmannin prevented AMPA receptor degradation (Shehata et al., 2012). In *C. elegans* neuromuscular system, internalized GABA receptors were reported to colocalize with autophagosomes (Rowland et al., 2006). Loss of both GABA and acetylcholine motor neuron innervations in the postsynapse, the dorsal muscle, caused internalization and sorting of only GABA, but not acetylcholine, receptors to autophagosomes in the dorsal muscle. This suggests that autophagy may employ cargo‐specific sorting for degradation postsynaptically.

### Canonical Endolysosomal Degradation

In addition to autophagy, neurons share canonical endolysosomal machinery with non‐neuronal cells. In endolysosomal degradation, membrane proteins are internalized through endocytosis and subsequently progress through late endosomal and lysosomal stages. Genetic studies in yeast, worms, flies, and mammals have identified a set of conserved and essential proteins that function in the endolysosomal progressions and lysosome biogenesis. The two ubiquitously expressed small Rab GTPases, Rab5 and Rab7, are key regulators of endocytosis and endosomal maturation, respectively [Fig. 2(B)] (Bucci et al., 1992; Vitelli et al., 1997). The ESCRT pathway is the core machinery that sort and direct ubiquitinated proteins for degradation (Katzmann et al., 2001; Henne et al., 2011; Schuh and Audhya, 2014). Membrane proteins can be ubiquitinated by the sequential action of E1 (ubiquitin‐activating enzymes), E2 (ubiquitin‐carrier/conjugating enzymes), and E3 (ubiquitin ligases). Ubiquitinated membrane proteins are then recognized by the ESCRT‐0 complex, which results in subsequent recruitment of ESCRT‐I, ‐II, and –III for delivery of the ubiquitinated protein into MVB by intraluminal vesicle (ILV) formation (Henne et al., 2011; Schuh and Audhya, 2014). Numerous regulatory and tethering complexes are required for endolysosomal progression, including the CORVET and HOPS, which are reviewed in detail elsewhere (Balderhaar and Ungermann, 2013; Solinger and Spang, 2013; Luzio et al., 2014).

Rab7 is expressed ubiquitously in all cell types of an organism, but its early expression is strongly enriched in the nervous system in *Drosophila* (Chan et al., 2011). Missense mutations in *rab7* cause the neuropathy Charcot–Marie–Tooth type 2B (CMT2B) in patients (Verhoeven et al., 2003). Studies of the disease‐associated mutations suggested toxic gain‐of‐function effects in cell culture (Spinosa et al., 2008; Cogli et al., 2010; McCray et al., 2010; Cogli et al., 2013; Zhang et al., 2013). In contrast, a study in *Drosophila* revealed no toxic effects of the mutant proteins, but indicated partial loss of function as the underlying mechanism (Cherry et al., 2013). It is intriguing that mutations in *rab7*, which is ubiquitously expressed, cause the first observable defect in some of the longest neurons in the human body. As discussed above, neurons may be more sensitive to defects in membrane protein degradation because of their longevity as well as morphological complexity. Hence, neurons, and particularly synapses, may have particularly high or specialized demands on endolysosomal degradation. However, it remains to be investigated what membrane proteins are degraded by canonical endolysosomal degradation as opposed to autophagy and neuron‐specific mechanisms in neurons (Fig. 2).

### Skywalker/Rab35‐Dependent Sorting and Degradation Is Cargo‐Specific

At axon terminals, synaptic vesicles undergo continuous exo‐/endocytic recycling (Heuser and Reese, 1973; Sudhof, 2004; Rizzoli, 2014; Kononenko and Haucke, 2015). Endocytosed membrane and membrane proteins are either sorted for synaptic vesicle recycling or degradation. Sorting stations have been proposed as “sorting endosome” or “early vacuoles.” Key regulators of this sorting mechanism are Rab35 and its GTPase activating protein (GAP), Skywalker [Fig. 2(B)] (Uytterhoeven et al., 2011; Sheehan et al., 2016). In *sky* mutants, Uytterhoeven et al. (2011) observed that synaptic vesicle recycling through the sorting endosome was increased. Correspondingly, degradation of dysfunctional (ubiquitinated) n‐Syb was increased, and this resulted in an increased readily releasable pool (RRP) and synaptic neurotransmission at the *Drosophila* NMJ. Mutations in *skywalker* were also reported to cause epilepsy and DOORS syndrome in human (Fischer et al., 2016). Using a fluorescence timer tagged n‐Syb, Fernandes et al. (2014) reported an increased protein turnover through lysosomal degradation in the *sky* mutant, which resulted in a younger pool of proteins compared to wild‐type (Fernandes et al., 2014). These seminal studies show how local synaptic vesicle protein turnover affects the pools of functional synaptic vesicles. Sorting and degradation may require recognition of dysfunctional proteins, as indicated by ubiquitination. Alternatively, the *sky‐*dependent mechanism may function to continuously turn over synaptic vesicle proteins independent of their functional status. It remains to be investigated whether and how functional and dysfunctional proteins residing on the same vesicle could be sorted out and whether their degradation ultimately occurs locally.

Sheehan et al. (2016) report the first evidence of differential specificities for different synaptic vesicle proteins through the Rab35‐dependent mechanism in vertebrate neurons. On neuronal activation, the Skywalker/Rab35 and ESCRT pathway selectively sorts and degrades neuronal Synaptobrevin (n‐Syb)/VAMP2 and SV2, but not Synaptotagmin 1 (Syt1) and Syntaxin (Syx). This suggests that individual SV proteins are recognized and sorted for degradation. How Sky/Rab35 recognizes these specific proteins for degradation remains an open question. It also remains unknown whether Sky/Rab35 specifically sorts SV proteins or also other non‐SV proteins in the presynaptic terminal, whether this sorting mechanism also exists in the postsynaptic terminals, and whether retrograde trafficking of degradative vesicles plays a role.

### Neuron‐Specific Endolysosomal Degradation

Autophagy and the Sky/Rab35‐dependent endolysosomal mechanism exist in all cells, but are likely to function in a specialized manner at synaptic terminals (Fernandes et al., 2014; Vijayan and Verstreken, 2017). In addition, at least two neuron‐specific integral membrane proteins function in endolysosomal sorting and degradation based on findings in *Drosophila*: the vesicle SNARE neuronal Synaptobrevin (n‐Syb) and the vesicle ATPase component V100 (Williamson et al., 2010a; Haberman et al., 2012). Both proteins have previously been characterized as synaptic vesicle proteins (Perin et al., 1991; Schoch et al., 2001). Loss of either protein in *Drosophila* photoreceptor neurons leads to endolysosomal membrane accumulations at axon terminals, indicating a link between the synaptic vesicle cycle and synaptic membrane turnover (Williamson et al., 2010a; Haberman et al., 2012; Wang and Hiesinger, 2012; Bezprozvanny and Hiesinger, 2013). However, it is currently unclear whether this neuron‐specific branch of the endolysosmal system has a cargo‐specificity that differs from the canonical endolysosomal degradation. Several plasma membrane receptors have been shown to accumulate in the *v100* mutant brains (Williamson et al., 2010b). In the *v100* mutant photoreceptor neurons, different membrane receptors accumulated in cell bodies versus axon terminals, suggesting that the neuron‐specific branch of the endolysosomal system may sort and degrade different membrane proteins in axon terminals versus in the cell body.

## WHEN ARE MEMBRANE PROTEINS DEGRADED IN NEURONS?

Membrane protein turnover plays roles during neuronal development, function, and maintenance. Constitutive membrane protein degradation may occur during all stages of a neuron's lifetime and is not regulated by neuronal activity [Fig. 2(A)]. In contrast, activity‐dependent turnover and degradation is a direct function of neuronal activity levels and is relevant to function and maintenance [Fig. 2(B)]. In this last section, we highlight the differences between these mechanisms during the life of a neuron.

### Constitutive and Developmental Degradation

Activity‐independent, constitutive protein degradation has been highlighted recently (Maday and Holzbaur, 2016; Cohen and Ziv, 2017). Synaptic vesicle proteins may undergo constitutive turnover and degradation already during development [Fig. 2(A)]. Synaptotagmin 1 (Syt1), a calcium sensor necessary for synaptic vesicle release, and n‐Syb are already present at axon terminals prior to synaptogenesis (Hiesinger et al., 1999; Williamson et al., 2010b). While synaptic autophagy has been reported as a constitutive degradation mechanism (Maday and Holzbaur, 2016), it remains unclear whether this mechanism degrades synaptic vesicle proteins during development.

Membrane protein degradation has been implicated in neural development, including axonal growth, synapse elimination, and pruning (Yogev and Shen, 2014; Wojnacki and Galli, 2016). Spatiotemporal control of developmentally required membrane receptor availability on the surface of axon terminals is regulated through protein turnover. Defects in the endolysosomal system lead to accumulations of undegraded membrane proteins before and after synaptogenesis (Wang et al., 2013). Mutations in the ubiquitous endosomal maturation factor *rab7* in *Drosophila* surprisingly do not affect embryo and larval development (Cherry et al., 2013). However, membrane proteins slowly accumulate during development and autophagy is upregulated as a consequence. During later adult stages, the impaired clearance of membrane proteins in the developing organism causes neurodegeneration. *Drosophila* photoreceptor neurons deficient for *rab7* complete development and function normally as long as they are not stimulated (Cherry et al., 2013). These findings indicate that constitutive *rab7*‐dependent endolysosomal turnover is not required for development of these cells. Similarly, mutations in the neuron‐specific genes *n‐Syb* and *V100* lead to accumulation of early endosomes and autophagosomes during development, but do not affect photoreceptor neuron development (Williamson et al., 2010a, 2010b; Haberman et al., 2012; Cherry et al., 2013). Timing may be critical: since *Drosophila* photoreceptor neurons develop over a timespan of only a few days, “debris” accumulation may not affect these fast developing neurons as profoundly as neurons with longer development.

Developmental elimination of excess synapses through pruning is reported to occur via autophagy. Components of autophagic machinery begin to express and localize to axons in early development (Song et al., 2008). Blocking autophagosome formation by *Atg7* knockdown causes overextension of axons, whereas activation of autophagy by rapamycin suppresses axonal extension (Ban et al., 2013). In developing motor neuron axon terminals, the NMJ, excessive synapses are eliminated through engulfment of retracting axon tips by the surrounding glial cells, and the degrading axonal membranes were associated with LC3‐positive autolysosomes (Song et al., 2008). On the postsynaptic site, hyperactivated mTOR, hence, impaired autophagy, has been linked to reduced developmental dendritic pruning causing autism‐like neurodevelopmental disorders. mTOR inhibition by rapamycin corrects developmental spine pruning defects in mice mutant for autism‐causing *Tsc2* (Tang et al., 2014).

### Activity‐Dependent Degradation

Activity levels of neurons have a substantial impact on the turnover rate of SV proteins and transmembrane receptors on both the presynaptic and postsynaptic side. The Sky/Rab35 mechanism is activity‐dependent, while a possible constitutive role has not yet been shown. As described above, sky was originally discovered in *Drosophila* (Uytterhoeven et al., 2011) and the Rab35 mechanism was recently shown to function activity‐dependently in rat hippocampal neurons as well (Sheehan et al., 2016; Sheehan and Waites, 2017). Sheehan et al. compared protein levels before and after treatment of cultured neurons with either activity enhancer or blocker. Neuronal activity induced Rab35 activation and binding to the ESCRT‐0 protein Hrs, which they identified as a novel Rab35 effector. Their findings demonstrate that the Rab35/ESCRT pathway facilitates the activity‐dependent removal of SV proteins, to maintain presynaptic protein homeostasis (Sheehan et al., 2016; Sheehan and Waites, 2017). It remains to be shown to what extent individual proteins or organelles are recognized as dysfunctional, or, alternatively, whether continuous turnover of proteins or organelles irrespective of their functional state may suffice to ensure neuronal health.

Prolonged neuronal activity also induces autophagy at both presynaptic and postsynaptic sites. Soukup et al. (2016) induced action potentials in motor neurons by the overexpression of transient receptor potential cation channel A1 (TrpA1) and observed increased formation of Atg8‐positive autophagosomes and LAMP2‐positive lysosomes at presynaptic terminals (Soukup et al., 2016). Conversely, neuronal stimulation in rat hippocampal neurons induces autophagosome formation both presynaptically and postsynaptically and possibly regulates the degradation of GABA and AMPA receptors (Shehata et al., 2012; Widagdo et al., 2015).

Activity‐dependent regulation of the density and number of AMPA and kainate receptors on the postsynaptic membrane is a key feature of long‐term changes on synaptic strength. Also, long‐term memory formation from unstable short‐term memory traces depends on rapid spatiotemporal changes of synaptic protein composition (Fioravante and Byrne, 2011; Jarome and Helmstetter, 2014). Activity‐dependent sorting of AMPAR to lysosomes has been reported (Ehlers, 2000; Schwarz et al., 2010; Widagdo and Anggono, 2015; Widagdo et al., 2015). More specifically, activity‐dependent ubiquitination of AMPAR results in sorting into LAMP1‐positive lysosomes for degradation (Ehlers, 2000; Schwarz et al., 2010; Widagdo et al., 2015). In a similar manner, intense activation of kainate receptors causes a PKC‐dependent, but Ca^2+^‐independent, internalization into lysosomes for degradation (Martin and Henley, 2004). Intriguingly, recruitment of lysosomes to dendritic spines was reported to be activity‐dependent (Goo et al., 2017). In sum, neuronal activity regulates the intracellular trafficking and degradation of postsynaptic membrane proteins to allow rapid spatiotemporal changes of synaptic protein composition, which has been implicated in long term memory formation (Fioravante and Byrne, 2011; Jarome and Helmstetter, 2014). However, it remains unclear in most cases, whether degradation occurs locally at the synapse or during and after retrograde trafficking back to the cell body.

## CONCLUSION

In this review, we highlighted recent advances and open questions on neuronal membrane protein degradation. Recent studies have beautifully shown that specific membrane proteins are sorted for degradation by different mechanisms, at different places in neurons and during different times. However, our survey of the *where, what*, and *when* of neuronal membrane protein degradation highlights key open questions:

1. *Where?* While local sorting has been demonstrated for all neuronal compartments, the question of local degradation versus retrograde trafficking remains largely unanswered.
2. *What?* The cargo‐specificity of both autophagy and endolysosomal degradation mechanism is largely unknown and may vary in the different neuronal compartments.
3. *When?* Both endolysosomal and autophagic degradation have been proposed as constitutive and activity‐dependent mechanisms in various contexts. When these mechanisms are activated in neurons will require experimental evidence from both *in vitro* and *in vivo* studies.

Answers to these questions are complicated by interdependencies: axonal and dendritic terminals are likely to employ different membrane sorting and degradation mechanisms as a function of developmental and functional stages. Conversely, the same mechanism, for example, autophagy, has been proposed to function differently, and possibly with altered cargo‐specificity, in different cellular compartments. Biochemical analyses of such spatiotemporally segregated and interdependent process are difficult and are dependent on technological advances such as microfluidic chambers and isolation of the different neuronal compartments. Primary neuronal culture is an excellent choice for cell biological studies using both biochemistry and imaging, but may not always reflect normal developmental and functional context. Finally, *in vivo* systems are typically less accessible. As always, a combination of these techniques is most likely to yield meaningful solutions, as long as each system is interpreted relative to other approaches and its own limitations.

We would like to thank all members of the Hiesinger lab for discussions.
